# The Role of the *Leishmania infantum* Infected Dogs as a Potential Reservoir Host for Toscana Virus in a Zoonotic Visceral Leishmaniasis Focus of Northern Tunisia

**DOI:** 10.3390/v15041012

**Published:** 2023-04-20

**Authors:** Khalil Dachraoui, Ifhem Chelbi, Imen Labidi, Raja Ben Osman, Aida Sayadi, Mourad Ben Said, Saifedine Cherni, Mohammed Abdo Saghir Abbas, Rémi Charrel, Elyes Zhioua

**Affiliations:** 1 Unit of Vector Ecology, Institut Pasteur de Tunis, Tunis 1002, Tunisia; 2 Laboratory of Transmission, Control and Immuno-Biology of Infections, Institut Pasteur de Tunis, Tunis 1002, Tunisia; 3 Vaccine Control Unit, National Drug Control Laboratory, Tunis 1002, Tunisia; 4 Department of Basic Sciences, Higher Institute of Biotechnology of Sidi Thabet, University of Manouba, Manouba 2010, Tunisia; 5 Laboratory of Microbiology, National School of Veterinary Medicine of Sidi Thabet, University of Manouba, Manouba 2010, Tunisia; 6 Unité des Virus Emergents, Aix-Marseille University, IRD 190, INSERM U1207, 13005 Marseille, France; 7 Laboratoire des Infections Virales Aigues et Tropicales, Pole des Maladies Infectieuses, AP-HM Hôpitaux Universitaires de Marseille, 13005 Marseille, France

**Keywords:** Toscana virus, reservoir, dogs, xenodiagnosis, *Phlebotomus perniciosus*, zoonotic visceral leishmaniasis, *Leishmania infantum*, transmission dynamics

## Abstract

The role of dogs as reservoir hosts for Toscana virus (TOSV) remains undetermined. This study investigated TOSV and *Leishmania infantum* infections in one healthy and three infected dogs with *Leishmania* (A, B, C) following natural exposition to sandfly bites in a focus of zoonotic visceral leishmaniasis (ZVL) located in Northern Tunisia from June to October 2020. At the end of the exposition period, infected and healthy dogs were examined for TOSV and *L. infantum* infections by xenodiagnosis using a colony of *Phlebotomus perniciosus*. Pools of freshly engorged *P. perniciosus* at days 0 and those at days 7 post-feeding were screened for TOSV and *L. infantum* by nested PCR in the polymerase gene and kinetoplast minicircle DNA, respectively. In the exposure site, *P. pernicious* is the most abundant sandfly species. The infection rates of sandflies with TOSV and *L. infantum* were 0.10 and 0.05%, respectively. *Leishmania infantum* DNA and TOSV RNA were detected in *P. perniciosus* females fed on dog B and C, respectively. The isolation of TOSV in Vero cells was achieved from two pools containing *P. perniciosus* fed on dog C. No pathogens were detected in *P. perniciosus* females fed on dog A and on control dog. We report for the first time the reservoir competence of dog with ZVL in the transmission of TOSV to sandfly vectors in natural settings, in addition to its role as a main reservoir host of *L. infantum*.

## 1. Introduction

*Phlebotomus perniciosus* is the main vector of *Leishmania infantum*, an etiological agent of zoonotic visceral leishmaniasis (ZVL) affecting humans and dogs in the Western Mediterranean basin [1,2]. *Phlebotomus perniciosus* is also the main vector of Toscana virus (TOSV) [3], a sandfly borne phlebovirus that exhibits neurotropism and may lead to aseptic meningitis and meningo-encephalitis in the Mediterranean basin [4].

Since both pathogens are transmitted by the same arthropod vector [1,2,3], human and dog populations living in Western Mediterranean countries are at high risk for *L*. *infantum* and TOSV infection. A serological study performed on randomly selected patients hospitalized between 2001 and 2010 in the Public Hospitals of Marseille without ZVL showed that humans could be co-infected by *L*. *infantum* and TOSV [5]. This double infection could be the result of co-infected or successive infecting bites of *P*. *perniciosus* with *L*. *infantum* and TOSV [5]. Similarly, co-infected dogs by *L*. *infantum* and TOSV were reported from Southeastern Turkey [6]. A geographical overlap between the distribution of ZVL and TOSV infection in humans and canines was observed in different bio-geographical areas of Tunisia [7,8,9,10,11], suggesting an epidemiological link between *L*. *infantum* and TOSV infections.

The domestic dog is considered as the main reservoir host for *L*. *infantum* in the Old World [12,13] and in the New World [14]. While the dog is the main reservoir of *L*. *infantum*, its role as a reservoir host for TOSV remains undetermined. Several studies performed on dogs from Mediterranean countries reported seroprevalence of TOSV up to 40% [6]. A seroprevalence of 7.5% of dogs for TOSV was reported in Tunisia, and subsequently, it has been suggested that dogs could be used as sentinels for monitoring the activity of this phlebovirus [9]. The co-circulation of *L*. *infantum* and TOSV in sandflies in the ZVL focus provides strong evidence that dogs are exposed to both pathogens following successive infected sandfly bites [15]. In ZVL foci with co-circulation of *L*. *infantum* and TOSV in sandflies, dogs are exposed to bites of infected *P*. *perniciosus*, the main vector of these two pathogens. Thus, we hypothesized that canines living in endemic ZVL foci are potential reservoir hosts for TOSV and *L*. *infantum*.

## 2. Material and Methods

### 2.1. Experimental Protocol

This study was performed in an endemic area for ZVL where *P*. *perniciosus* is the most abundant sandfly species, followed by *P*. *perfiliewi* [16]. The study took place from June to October, 2020, a period corresponding to the main peak of activity of *P*. *perniciosus* and *P*. *perfiliewi* [17,18], at a rural dog kennel (36°58′ N, 10°03′ E) licensed by the Department of Agriculture and belonging to the governorate of Ariana in Northern Tunisia. It is of major epidemiological importance to point out that the study site is located in a ZVL endemic area [1,13].

### 2.2. Seasonal Activity of Sandflies in the Exposition Site

The phenology of sandfly species was studied during the same period in the same site. Sticky traps (20 × 20 cm) soaked in castor oil were used to collect sandflies. Thirteen sticky traps (total surface is 1 m^2^) tied with one cord and evenly spaced were suspended 2 m above the ground in one sheep pen. One trapping unit (13 sticky papers) was placed on a weekly basis for one night from June to October 2020. All collected sandflies by sticky traps were dead. Sandflies were removed from the sticky traps the following day with a fine-haired brush and placed in 95% ethanol, then mounted on glass slides in Mark André medium and identified to the species level using the identification keys of Croset et al. (1978) [19]. Density of sandflies was recorded as the number of sandfly species per m^2^ of sticky trap [17,18].

### 2.3. Collection, Identification, and Infection Prevalence of Wild Sandflies with L. infantum and TOSV in the Exposure Site

Concomitantly with dog’s exposition, the detection of *Leishmania* parasite and phlebovirus in wild sandflies was studied during the same period (from 11 June to 3 November 2020) in the same site. Sandflies were collected by using CDC Miniature Light Traps (John W. Hock Company, Gainesville, FL, USA) modified using an ultrafine mesh placed on a 20 × 20 cm cubic steel frame. The CDC light traps were placed inside the boxes where dogs were kept. Collected sandflies were pooled with a maximum of 30 individuals per pool according to trapping origin and sex, and were placed in 1.5 mL tubes, and stored at −80 °C until use. 

Taking into account that living sandflies collected by CDC light traps were used for viral and *Leishmania* parasite detection, their abundance was determined based on collected dead specimens found in the CDC cage. Identification of sandflies to the species level was performed on field-collected dead specimens according to morphological characters, with special attention to the atypical form of female *P*. *perniciosus*, which could be confused with *P*. *longicuspis* [19,20,21,22].

Pools of sandflies previously stored at −80 °C were processed as previously described [15]. Pools of female sandflies (with up to 30 females for pool) were homogenized using the automated Tissue Lyser LT (Qiagen, Hilden, Germany) with glass beads at a frequency of 50 cycles s^−1^ for 5 min. The mixture was clarified by centrifugation at 6000× *g* for 2 min to be used for DNA extraction (from 200 µL supernatant) with Qiagen DNA Mini Kit (Qiagen). The extracted DNA was screened for *L*. *infantum* infections by nested PCR of a partial region of the ITS-rDNA gene as previously described [23,24]. The first amplification steps were performed using the Taq DNA recombinant polymerase kit (Invitrogen, Waltham, MA, USA) in 50 µL of reaction containing: 5 µL 10× buffer, 3 µL MgCl_2_ (50 mM), 2 µL dNTP mix (10 mM), 1 µL of each reverse and forward primers IR1/IR2 (10 µM), 0.5 µL Taq DNA polymerase enzyme, and 10 µL of total extracted DNA. Nested PCR was carried out in 50 µL containing 2 µL of the first PCR step DNA product and 48 µL of mixture containing: 5 µL 10× buffer, 3 µL MgCl_2_ (50 mM), 2 µL dNTP mix (10 mM), 1 µL of each reverse and forward internal primers ITS1F/ITS2R4 (10 µM), and 0.5 µL of Taq DNA polymerase (Invitrogen, Waltham, MA, USA). Optimized cycling conditions for the first and second PCR steps were performed as follows: (*i*) first PCR with 94 °C for 3 min followed by 40 cycles of 94 °C for 60 s, 58 °C for 60 s and 72 °C for 90 s, followed by a final extension step (72 °C) for 10 min; (*ii*) nested PCR with 94 °C for 3 min followed by 5 cycles of 94 °C for 60 s, 55 °C for 60 s and 72 °C for 60 s, and 35 cycles of incubation at 94 °C, 59 °C and 72 °C for 60 s each. The extension step was continued for 10 min at 72 °C. Cross-contamination was monitored by negative controls for sample extraction and PCR solutions for PCR testing. The nested PCR amplification products were separated in a 2% agarose gel stained with ethidium bromide and visualized under UV-light illumination. The nested PCR products, having a size of 462 bp, were purified by the ExoSAP-IT method by using the Exonuclease-I and the Shrimp Alkaline Phosphatase and were sequenced in both directions by using a Big Dye Terminator ready reaction cycle sequencing v3.1 kit (Applied Biosystems, Waltham, MA, USA) with forward and reverse nested PCR primers (ITS1F/ITS2R4) [25].

In addition, total RNA extraction was performed on 200 µL female and male sandfly supernatant using the viral RNA Mini Kit (Qiagen). The presence of TOSV in the extracted RNA was performed by nested-reverse transcription PCR targeting the polymerase gene as previously described [26]. The reaction was carried out by using the SuperScript^®^ III One-Step RT-PCR System with Platinum^®^ Taq DNA Polymerase kit (Invitrogen) [11]. Amplification products of the nested PCR were then visualized by electrophoresis in a 2% agarose gel stained with ethidium bromide. In order to identify the presence of TOSV, specific reverse primers retested the first PCR product of positive pools for TOSV (Atos 2−) [26]. Nested positive PCR products were purified by ExoSAP-IT method [11], and sequenced using a Big Dye Terminator ready reaction cycle sequencing v3.1 kit (Applied Biosystems) with forward and reverse nested PCR primers (Nphlebo 2+/2−) [26].

The minimum infection rate (MIR) of sandflies by phlebovirus or *Leishmania* parasites is defined as the number of positive pools divided by the total number of tested specimens ×100 [27].

### 2.4. Ethics Statement

The maintenance of dogs and the experimental procedures used in this research program followed the Animal Care and Use Protocol, which is approved by the Institutional Animal Care and Use Committee of the Institut Pasteur de Tunis, Tunisia (2018/01/I/ES/IPT/V0). The Institut Pasteur de Tunis complies with the European Directive for the Protection of Vertebrate Animals used for experimental and other scientific purposes (2010/63/EU).

### 2.5. Xenodiagnosis

We used three Beagle dogs (one male and two females, aged from three to four years old) that had been naturally infected with *L. infantum* as part of a different study to investigate the efficacy of a vaccine against canine ZVL. These dogs had been exposed during the study period from September to November 2018 to wild sandfly bites under natural conditions in a ZVL focus located at Borj Youssef in the governorate of Ariana (36°57′ N, 10°05′ E) and were from the unvaccinated (control) group. Infection status of the infected dogs was confirmed by indirect immunofluorescent antibody test (IFAT), as previously described [13]. Samples that displayed a bright-green peripheral stain with a dull fluorescence of the parasite cytoplasm at 1:160 (cutoff point) were considered positive [13]. Our main hypothesis was to show that canines living in a ZVL endemic focus are potential reservoir hosts for TOSV. To test this hypothesis, xenodiagnosis as the golden standard needs to be used [13]. Thus, we did not foresee to assess the infection status of dogs for TOSV with serology and/or PCR neither at the entry of the study, nor at the end of the exposition period. The dogs were kept in the kennels of Institut Pasteur de Tunis after the exposure period in 2018 and showed clinical signs 11 to 14 months after being exposed to wild sandfly bites. All symptomatic dogs showing specific signs of ZVL including lymphadenomegaly, hepatomegaly, splenomegaly, and progressive weight loss were classified as infected when introduced into our study. In addition, one uninfected Beagle (one male, three years old) from the kennel of the Institut Pasteur de Tunis was used as a control. Infected dogs were housed individually in one part of the dog kennel and they were under veterinary care. Uninfected dog were housed in a separate part of the kennels. Protective measures were taken to avoid the infection of the uninfected dog as described previously [13]. Infected and uninfected dogs were housed in separate parts of the kennels in the exposure site where they received daily regular veterinary care. It is of major epidemiological importance to point out that xenodiagnosis is the most accurate method to ascertain the infectiousness of a reservoir host to sandfly vectors. Compared to ZVL, the infection status of dogs for TOSV was not assessed by serology and/or PCR. The reservoir competence of dogs for TOSV was ascertained only by xenodiagnosis.

Sandflies used in this study are from a colony originated from Tunisia and maintained at the Unit of Vector Ecology in the Institut Pasteur de Tunis since 2003 [28,29]. The three symptomatic dogs (A, B, C) and the uninfected Beagle dog used as a control have been exposed to wild sandfly bites under natural conditions in this ZVL focus from June to October 2020, a period corresponding to sandflies’ activity season [17,18]. At the end of sandfly season, corresponding to the end of the exposure period, all dogs were housed again at the kennel of Institut Pasteur de Tunis. 

On day 1 post exposure, the three symptomatic dogs and the healthy dog were anesthetized by intramuscular (IM) injection of a mixture of 1.5 mL of ketamine (15 mg/kg) (Merial, Lyon, France) and 0.02 mL/kg of acepromazine maleate (Kela, N.V., Hoogstraten, Belgium). Since ear skin is the best predictor of vector infection [30], the head of each dog was placed separately in a mesh cage (wire frames = 40 × 40 cm) for 80 min. A minimum of 60 females and 20 males uninfected laboratory-colonized *P*. *perniciosus* (F 98) were then released inside each cage. Male sandflies were present to stimulate the engorgement of females [28,29]. Unfed sandflies were 5–7 days old and deprived of sugar for 24 h before being used in this experiment. The work was performed in a bio-safety level laboratory 2. All sandflies were engorged at day 0 on dogs. Engorged sandflies from each dog at day 0 were subdivided equally into two batches of 30 specimens each. The first batch was sacrificed at D0. The second batch was kept in the insectarium until day 7 post-blood feeding before being sacrificed. All sandflies were examined for the presence of TOSV and *L*. *infantum* by PCR. Concomitantly, colonized *P*. *perniciosus* (F 98) were tested for the presence of TOSV and *L*. *infantum* by PCR to ascertain their negative status.

#### 2.5.1. Detection of *L. infantum* Infections in *P. perniciosus* Females Fed on Dogs

Pools of 30 engorged *P*. *perniciosus* females on the three symptomatic dogs and the one healthy dog were homogenized through high-speed shaking using the automated Tissue Lyser LT (Qiagen, Hilden, Germany) with glass beads. The mixture was clarified by centrifugation at 6000× *g* for 2 min. Total DNA extraction was performed on 200 µL supernatant using a Qiagen DNA Mini Kit (Qiagen, Hilden, Germany) according to the manufacturer’s instructions. Extracted DNA was screened for infections of *L*. *infantum* by a nested PCR of a partial region of the ITS-rDNA gene [23,24] as previously described for wild sandflies collected in the exposure site (Section 2.3).

#### 2.5.2. Detection of TOSV in *P. perniciosus* Fed on Dogs

Pools of 30 *P*. *perniciosus* fed on the three symptomatic dogs and the one healthy dog were processed as previously described [11,27]. Briefly, after sandfly grounding in enriched minimal essential medium (MEM), the mixture was clarified by centrifugation at 5800× *g* for 10 min. Total RNA extraction was performed on 200 µL supernatant using the viral RNA Mini Kit (Qiagen). The presence of TOSV in the extracted RNA was performed by nested-reverse transcription PCR in the polymerase gene as previously described [26]. The reaction was carried out by using the same primers used for wild sandflies collected in the exposure site (Section 2.3).

#### 2.5.3. Isolation of Phlebovirus from Infected *P. perniciosus* with TOSV following Engorgement on Dogs

Sandfly homogenates of TOSV-PCR positive samples were inoculated into Vero cell culture (ATCC CCL-81). To avoid cytotoxicity and contamination risk, a negative control was used. Three passages were performed with the Vero cell line. Between each passage, cells were monitored daily for 7 days for cytopathic effect (CPE), and 400 µL supernatant medium was collected for nucleic acid testing by RT-PCR using specific TOSV primers [26] after viral RNA extraction, as mentioned above. Flasks with CPE will be used to titrate the virus. To determine the TCID_50_ (Median Tissue Culture Infectious Dose) for the quantitation of virus infectivity, the virus titer was calculated by the reference method of Carrber [31].

### 2.6. Phylogenetic Analysis

For *L*. *infantum* and TOSV, the resulting consensus sequences were deduced by aligning the respective forward and reverse sequences using CLUSTAL_W 1.4 implemented in MEGA v.5.22 [32]. Phylogenetic analysis was carried out by using the maximum likelihood analysis method and the Tamura-3 parameter model. The trees’ topology was supported by 1000 bootstrap replicates. The homology and the nucleotide distances between the different sequences obtained for TOSV were calculated using the *p*-distance method within MEGA5.

## 3. Results

### 3.1. Abundance and Seasonal Activity of Sandflies in the Exposure Site

A total of 7024 sandflies were collected from the exposure site during the study period. Among these sandflies, 6211 were alive and, subsequently, they were pooled without identification and tested for the presence of *L*. *infantum* DNA and TOSV RNA. The remaining dead sandflies collected from the CDC cage were identified to the species level and, subsequently, the abundance was determined. A total of 813 dead sandflies collected by using CDC light traps were identified (289 females and 524 males) (Table 1). *P*. *perniciosus* was the most abundant species (41.94%), followed by *P*. *perfiliewi* (31.36%), *Sergentomya minuta parotti* (26.19%), *P*. *papatasi* (0.36%), and *P*. *longicuspis* (0.12%) (Table 1).

**Figure 1:**
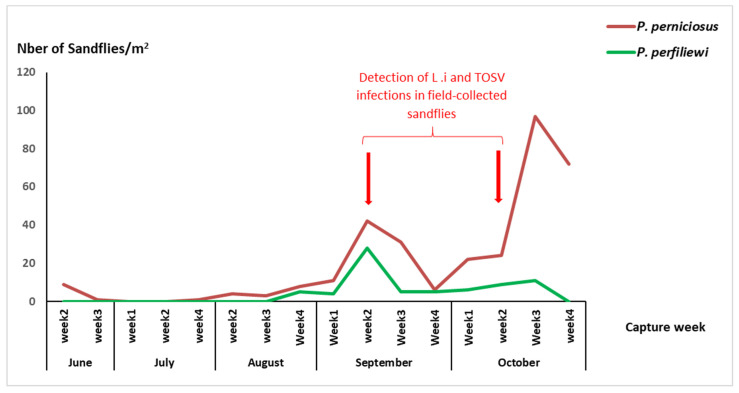
Phenology of sandflies of the subgenus *Larroussius*, collected in the exposure site, 2020 (L. i: *L. infantum*, TOSV: Toscana virus).

Collected sandflies by sticky traps on a weekly basis during the study period were identified to species level. The phenology of sandflies of the subgenus *Larroussius*, including *P*. *perniciosus* and *P*. *perfiliewi* showed two main peaks: a small one in June and a second larger one in September–October (Figure 1).

### 3.2. Detection of Leishmania infantum and TOSV in Field-Collected Sandflies from the Exposure Site

Field-collected sandflies brought alive to the laboratory were tested for *L*. *infantum* and TOSV without morphological identification to the species level, but they were only sorted based on sex and trapping date. This approach is less time consuming and allowed the processing of a large number of sandflies with reduced handling time, which is critical for preserving infectivity and increasing the chance of virus detection. A total of 6211 living sandflies (3730 females and 2481 males) were captured from June to October 2020 and pooled according to trapping origin and gender, with a maximum of 30 individuals per pool. A total number of 229 pools were obtained. Among them, 139 and 90 pools contained female and male sandflies, respectively. The collected sandflies were processed and tested for the presence of *L*. *infantum* and TOSV. The Minimum infection rates (MIRs) of sandflies with *L*. *infantum* and TOSV were 0.05% (2/3730) and 0.10% (6/6211), respectively (Table 2). TOSV-positive pools were detected in both female and male sandflies (Table 2). During this study, no co-infected pool was obtained. It is important to note that *L*. *infantum* and TOSV were detected during the second main peak of sandfly activity (September and October) (Table 2, Supplementary Table S1, and Figure 1).

### 3.3. Infection of Phlebotomus perniciosus with Leishmania infantum by Xenodiagnosis

All symptomatic dogs were seropositive, whereas the control dog was seronegative for *L*. *infantum*. Following the feeding of colonized *P*. *perniciosus* on the three symptomatic dogs A, B and C, a total of six pools were formed. Among these pools, three contained freshly engorged *P*. *perniciosus* at day 0 post-feeding (A_0_, B_0_ and C_0_), and three contained *P*. *perniciosus* pooled at day 7 post-blood feeding (A_7_, B_7_ and C_7_). Following the feeding of colonized *P*. *perniciosus* on the healthy dog, two pools were obtained of fed females *P*. *perniciosus*. All pools were screened for *L*. *infantum* infection by the nested-PCR-based schizodeme method targeting the partially conserved region of the kinetoplast minicircle DNA. Two pools were found to be positive for *L*. *infantum* DNA: one pool of *P*. *perniciosus* females fed on dog B at day 0 (B_0_), and one pool of *P*. *perniciosus* females fed on dog B screened at day 7 post-feeding (B_7_) (Table 3). Nevertheless, no *L*. *infantum* was detected in colonized *P*. *perniciosus* from the insectary. 

### 3.4. Infection of Phlebotomus perniciosus with Toscana Virus by Xenodiagnosis

All 8 pools of *P*. *perniciosus* fed on dogs, previously analyzed for *L*. *infantum* infection, were also screened for TOSV infection by nested RT-PCR. Two pools, corresponding to females *P*. *perniciosus* fed on dog C at day 0 post-blood feeding (C_0_) and at day 7 post-blood feeding (C_7_), were found to be infected with TOSV (Table 3). Nevertheless, no TOSV RNA was detected in colonized *P*. *perniciosus* from the insectary. Vero cells (ATCC CCL-81), inoculated with pools containing homogenates of *P*. *perniciosus* fed on dog C at day 0 post-blood feeding (C_0_) and at day 7 post-blood feeding (C_7_), showed a cytopathic effect (CPE), which was reproduced during the three serial passages from P1 to P3. The CPE was observed after 4 days for the first two passages, P1 and P2, and after 2 days for P3. The presence of TOSV was confirmed by positive reverse transcriptase PCR (RT-PCR) at each passage. Calculation of TCID_50_ performed by the Carrber’s reference method [31] revealed a high TOSV viral titer of 10^8.8^ tissue-cultured infecting dose of 50% of Vero cells per mL (TCID_50_/mL).

### 3.5. Phylogenetic Study

#### 3.5.1. Phylogenetic Analysis of *L. infantum*

**Figure 2:**
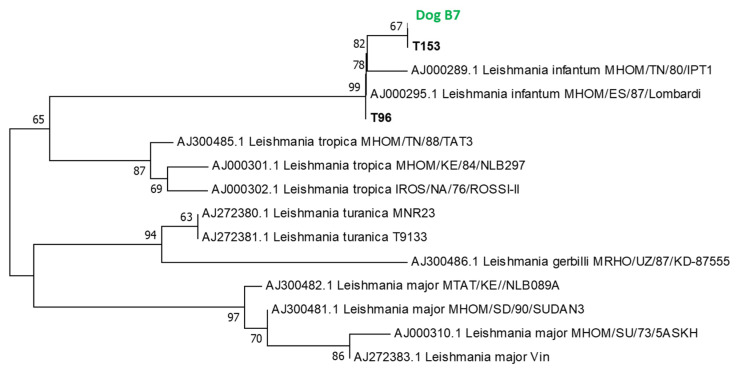
Phylogenetic tree based on partial *Leishmania* ITS-rDNA 5.8 s sequences.

All PCR products were sequenced, except for dog B_0,_ which contains *P*. *perniciosus* fed on dog B at day 0 post-blood feeding (day 0). Identified *Leishmania* DNA sequences are closely related to the reference sequence of *L*. *infantum* AJ000289 from Lombardi and *L*. *infantum* MHOM/TN/80/IPT1 isolated from Tunisia in 1980. Phylogenetic analysis showed that all *Leishmania* sequences (Dog B_7_, T96 and T153 corresponding to GenBank accession numbers OP788110, OP788108 and OP788109, respectively) clustered together with the *L*. *infantum* reference sequence. The phylogenetic branch including *L*. *infantum* sequences was supported by a high bootstrap value estimated at 99% (Figure 2).

#### 3.5.2. Homology, Genetic Distances and Phylogenetic Analysis of TOSV

TOSV sequences detected in *P*. *perniciosus* fed on dog C at day 7 post-blood feeding (C_7_) and those detected in wild sandflies were aligned. Homology and pairwise distances of the partial nucleotide sequences of the polymerase gene among the TOSV sequence detected in females *P*. *perniciosus* fed on dog C at day 7 post-blood feeding (C_7_) and the six TOSV sequences (T99, T114, T117, T139, T145 and T173) detected in field-collected sandflies are shown in Table 4. The homology and the nucleotide pairwise distances between the TOSV sequence detected in *P*. *perniciosus* fed on dog C_7_ and the six TOSV sequences T99, T114, T117, T139, T145 and T173 were 99.5/0.005, 100/0.000, 99.1/0.009, 100/0.000, 100/0.000 and 100/0.000, respectively (Table 4). These results clearly show that the TOSV strains circulating in the sandflies of pools T114, T139, T145 and T 173 are the same as the strain isolated from dog C by xenodiagnosis. However, the two strains circulating in the sandflies of pools T99 and T117 are closely related but slightly distinct from the strain isolated from dog C by xenodiagnosis.

**Figure 3:**
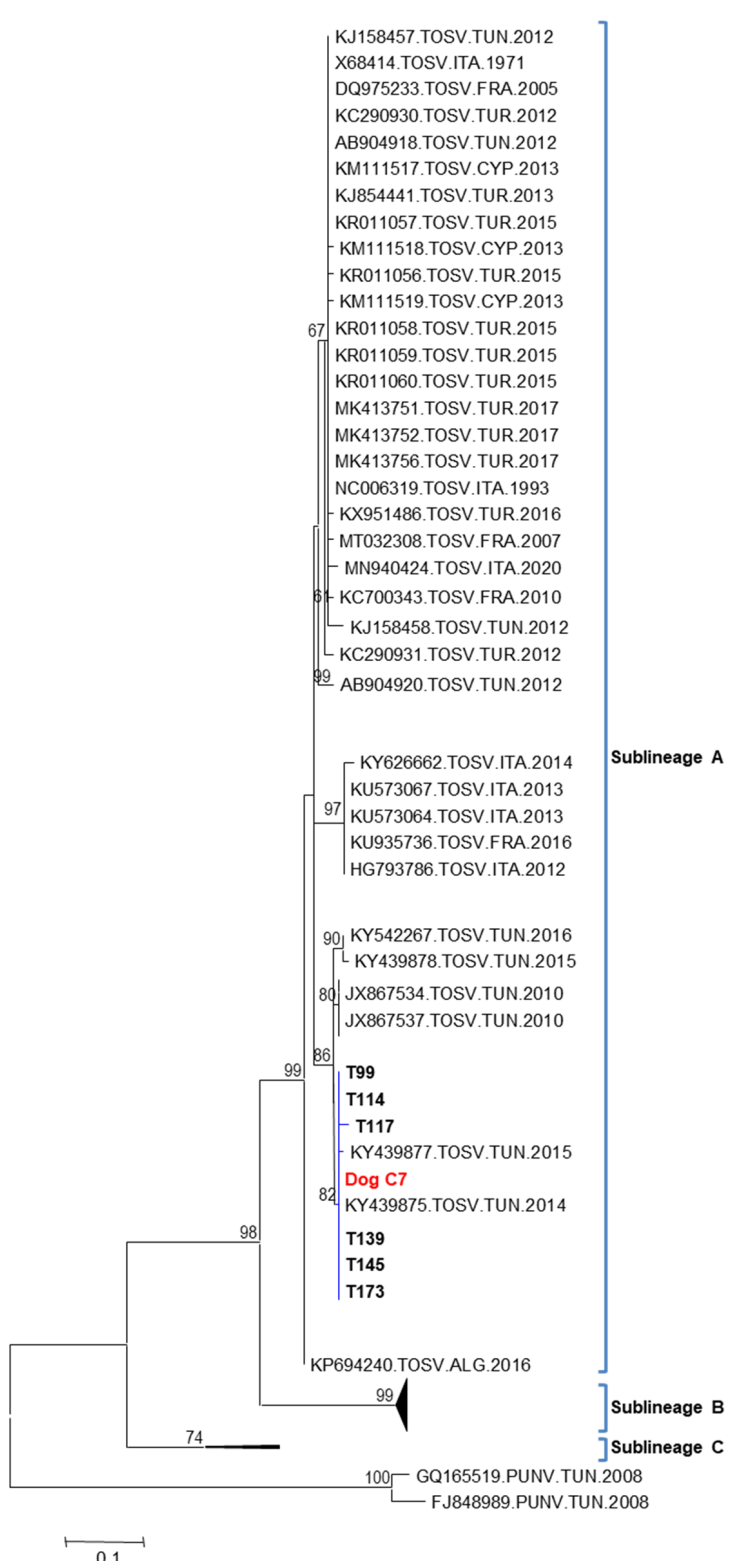
Phylogenetic tree based on the 201 bp of the L segment. Phylogenetic analysis was performed using the maximum likelihood analysis method and the Tamura 3 model. The tree topology was supported by 1000 bootstrap replicates. Punique virus (PUNV) sequences were used as an out-group.

All PCR products except for dog C_0_ obtained using primers located in the polymerase (gene L RNA segment), were cloned and sequenced, giving partial sequences of 201 bp. The phylogenetic tree based on partial nucleotide sequences of the polymerase gene showed that TOSV sequences identified in this study and those previously described in 2010, 2014, 2015 and 2016 were grouped together in sublineage A within a single phylogenetic cluster with a high bootstrap value estimated at 86% (Figure 3). Although the sequence used for phylogenetic analysis is short, (*i*) many studies have been based on this short region of the genome and therefore they allow us to gather the largest genetic diversity of TOSV sequences, (*ii*) the high values of the bootstrap analysis support that the clustering is solid (Figure 3), and (*iii*) the detailed analysis of the alignment showed that there is an important variability between the selected sequences that support the subclustering in five groups within lineage A. Of course, the shortness of the region does not allow us to infer elaborated phylogenetic conclusions, but we believe they adequately reflect the genetic diversity observed between strains from different countries and support the conclusions of our study. It is important to note that TOSV sequences identified in this study were not grouped with those previously reported in 2012 from Tunisia (Figure 3). GenBank accession numbers corresponding to the seven TOSV sequences detected in our study are OP793856 (dog C_7_), OP793850 (T99), OP793851 (T114), OP793852 (T117), OP793853 (T139), OP793854 (T145), and OP793855 (T173).

## 4. Discussion

The entomological surveillance performed during the study period showed clearly that sandfly species of the subgenus *Larroussius*, mainly *P*. *perniciosus*, and *P*. *perfiliewi*, were the most abundant species in this ZVL focus. The phenology of *P*. *perniciosus* and *P*. *perfiliewi* is characterized by two peaks: a small one in June and second larger later one in the fall as has been reported previously [18,33]. These two sandfly species are the main vectors of *L*. *infantum* and TOSV in the Western Mediterranean basin including Tunisia [11,34], and subsequently, the co-circulation of these two pathogens is expected in sandfly vector populations within this ZVL focus. 

In this study, we reported MIRs for TOSV and *L*. *infantum* 0.10% and 0.05%, respectively. We had previously conducted an entomological investigation in Central Tunisia over three years (2014 to 2016) [15]. The MIRs of sandflies with TOSV and *L*. *infantum* for 2014, 2015, and 2016 were 0.05%/0.25%, 0.11%/0.12%, and 0.22%/0.79%, respectively [15]. Several studies performed in the Mediterranean countries reported co-circulation of phleboviruses and *L*. *infantum* in ZVL endemic areas. Phleboviruses distinct from TOSV were detected in *P*. *perniciosus* collected from a ZVL focus of south-west of Madrid region, Spain [35]. *Leishmania infantum* and Massilia virus (MASV) were detected in field-collected *P*. *perniciosus* from the same site in Southern France [36]. Co-circulation of TOSV and *Leishmania tropica* in *Phlebotomus sergenti* was reported from a cutaneous leishmaniasis focus located in Central Morocco [37]. Co-infection of TOSV and *L*. *infantum* was characterized in one pool of *Phlebotomus tobbi* collected from Northern Cyprus [38]. Our results provide strong evidence that ZVL foci in Tunisia are primarily characterized by: (1) the predominance of sandfly species of the subgenus *Larroussius*, mainly *P*. *perniciosus* and *P*. *perfiliewi*, and (2) the co-circulation of TOSV and *L*. *infantum*, leading to closely epidemiological relationships between ZVL and phleboviral infections in humans and canines [5,6].

In the present and previous studies, we showed that male sandflies were infected with TOSV, suggesting transovarial and veneral transmission, and therefore, sandflies act as a reservoir hosts in addition to their role as a vector of this phlebovirus [11,15,27,33]. However, it was shown experimentally that the mechanism of transovarial transmission alone is not able to maintain viral transmission over five or six generations of sandflies, suggesting the existence of reservoir hosts needed for a prolonged maintenance of TOSV in nature [39,40,41]. Several domestic and wild animal species including bats were suspected to act as amplifying hosts for TOSV, but no evidence of infection was identified [3,42]. Compared to *L*. *infantum*, the role of dogs as reservoir hosts for TOSV remains undetermined. 

Despite the direct inoculation of TOSV at a higher dose (10^7^ TCID_50_) and at a low dose (10^4^ TCID_50_) to healthy dogs, none of them developed either clinical sign of infection or detectable neutralizing antibodies, and subsequently, healthy dogs were not considered as reservoir hosts for TOSV [43]. Consequently, dogs do not play a significant role in the transmission cycle of this sandfly-borne phlebovirus [43]. Nevertheless, the seroprevalence of TOSV in canines reported from several Mediterranean countries suggests that dogs are potential reservoirs [6,9]. In addition, the co-infection with *L*. *infantum* and TOSV reported among dogs with symptoms of canine leishmaniasis in Southeastern Turkey suggest that infected dogs with *L*. *infantum* are a potential reservoir for TOSV [6]. Infected dogs with *L*. *infantum* are highly attractive to *P*. *perniciosus* compared to healthy ones, and therefore, they act as sandfly magnets in a natural setting, allowing a significant increase of the contact between the vector and infected dogs [1]. Consequently, canines with symptoms of ZVL may contribute to an active replication of the virus and play a role in the maintenance of TOSV in nature. Thus, in the present study, *L*. *infantum*-infected dogs were examined for their infectivity of TOSV to sandflies.

Viremia levels following infections of humans or other vertebrates with phleboviruses are transient [43,44]. In order to confirm the role of dogs as reservoir hosts, and subsequently their impact on the maintenance of TOSV in nature, the experimental transmission of high viral loads to colonized sandflies in controlled environment must be demonstrated. Xenodiagnosis performed on canine B with symptoms of ZVL that was exposed to wild sandflies in a ZVL focus, revealed the transmission of *L*. *infantum* to naïve *P*. *perniciosus* females as it has been showed in our previous study [13]. This finding provides further evidence of the role of dogs in the natural transmission of *L*. *infantum*, an etiologic agent of ZVL [13]. It is important to point out that two out of three infected dogs did not transmit *L*. *infantum* to sandflies. These results are in agreement with previous studies showing that only 60% of dogs with clinical symptoms were infectious to sandflies [45]. More importantly, following the blood-feeding of naïve *P*. *perniciosus* females on infected canine C with symptoms, which was exposed to wild sandflies in a ZVL focus, TOSV was detected at D_0_ and D_7_ post blood-feeding, causing CPE on Vero cells, with a viral titer of 10^8.8^ TCID_50_/mL after three serial passages from P1 to P3. Two possible scenarios should be considered. First, it is quite possible that dog C became infected at the end of the exposition period and was TOSV viremic at D0, and subsequently infectious to the sandfly vector. In this case, the reservoir role for *L*. *infantum* infected dogs would be reconsidered and it would only be demonstrated that viremic dogs are capable of infecting vectors with TOSV. However, the direct inoculation of TOSV at a higher dose (10^7^ TCID_50_) and at a low dose (10^4^ TCID_50_) to healthy dogs, none of them developed either clinical sign of infection or detectable neutralizing antibodies, and subsequently, healthy dogs were not considered as reservoir hosts for TOSV [43]. Secondly, dog C encountered intermittent transient viremia during the peak of circulation of TOSV in sandflies (Figure 1), leading to a cyclic infectiousness status of *L*. *infantum*-infected dogs to sandfly vectors during the transmission season. This may explain the absence of infectiousness of dogs A and B for TOSV to sandflies. It is important to point out that we did not foresee to check the infection status of dogs with serology and/or PCR for TOSV, and subsequently, we are aware that it may represent a weakness in the experimental design. Thus, investigating these two scenarios with a larger number of dogs involving a follow-up of the kinetics of TOSV viral loads and serology is of major importance for a better understanding of the transmission dynamics of TOSV.

While TOSV was detected at a high viral load after three serial passages, the real infectious viral title in the dog remained undetermined. However, the detection of TOSV at a high viral load in sandflies just after day 0 post-blood feeding on canine with ZVL and on day 7 after blood digestion provide strong evidence that the phlebovirus was able to overcome several barriers within the vector to become infectious. Thus, we report for the first time that a dog with symptoms of ZVL is a competent reservoir host for TOSV and its role in the natural transmission cycle of this phlebovirus in nature. Although this finding was observed in only one out of four dogs, three of which were suffering from ZVL, it is of great interest when comparing with the previous study of Munoz et al. [43], reporting that healthy dogs are unlikely reservoir hosts and do not appear to play a significant role in the natural transmission cycle of TOSV. We hypothesized an epidemiological link between the infection of dog C with *L*. *infantum* and its infectiousness to *P*. *pernicious* for TOSV. Whether the infectiousness of dog C for TOSV to sandflies is independent or not of its *L*. *infantum* infection status deserves further investigation. Whether this situation plays a role in the natural transmission and maintenance of TOSV in a given environment remains to be quantified and studied in a more detailed and extended manner. It is of major importance to point out that circulating TOSV in this ZVL focus were detected only during the second peak of the activity of sandflies, suggesting a build-up process of infection between sandflies and dogs as amplifying hosts starting when adult emerge in the first peak of activity during spring to reach a peak of infection late in autumn in a similar way of *Leishmania* infection in sandflies [17].

It is important to note that neither canine A nor the healthy control dogs transmitted either pathogens to naïve *P*. *perniciosus* females. Only canine B transmitted *L*. *infantum*. The fact that dog C was viremic just after being extracted from the exposure site can be explained: either dog C was infected very shortly before exposure was stopped; or dog C was infected when sandfly density started to increase (from Sept); during this latter period, TOSV was detected twice when the density of *P*. *perniciosus* was 40 and 20 specimens per m², respectively. According to the latter, dog C would have endured prolonged viremia for weeks or even months, a situation that has never been reported so far; however, this is the first time, to the best of our knowledge, that dogs with ZVL have been tested after prolonged exposure to sandfly populations showing repeated TOSV circulation. Still, in this latter, and according to the previously described data, the immune status of the dog affected with leishmaniasis is likely to have played an important role. A study, specifically dedicated to investigating the ability of dogs affected with leishmaniasis to act as reservoirs for TOSV, is currently being planned with a greater number of dogs and involving follow-up of the kinetics of TOSV viral loads and serology together with markers for immunity monitoring.

Infections with *L*. *infantum* in immune competent adults remain mostly non-symptomatic. However, in young children, in immune compromised adults and rarely in healthy adults, the infection may progress into generalized parasite amplification in the reticulo-endothelial system, resulting in severe immune pathologies in spleen, liver and bone marrow. In the absence of an effective vaccine, treatment and control of ZVL depends solely on chemotherapy. If left untreated or if misdiagnosed, ZVL mostly has a fatal outcome [46]. It is not known currently which factors may trigger disease progression rather than control of the infection. A known compounding factor is the co-infection with HIV, which can result in the reactivation of previously latent *Leishmania* infections and which mostly precludes a successful chemotherapy [47]. Taking into account that both TOSV and *L*. *infantum* are transmitted in Tunisia mainly by *P*. *perniciosus*, phleboviral infections may modulate ZVL development in humans as well as in dogs. This hypothesis is corroborated by (1) the closely epidemiological relationships between infection by TOSV and ZVL in humans and in dogs [5,6,9], and (2) the recent findings showing that phleboviral infection contributes significantly in promoting *Leishmania* infection [48,49,50,51].

ZVL and human infection with TOSV represent serious public health problems in Tunisia [7,9,10]. Thus, understanding the transmission dynamics of TOSV is of major epidemiological importance and represents a cornerstone in the development of control approaches. A high prevalence of *L*. *infantum* and TOSV in dogs as the main reservoir host for these two vector-borne diseases is a key factor for triggering transmission to humans and to canines. Consequently, canines are an important parameter in the control of ZVL and TOSV and their transmission [52].

## Figures and Tables

**Table 1:** Abundance of sandflies collected by CDC light traps in the exposure site, 2020.

Species	Number of Sandflies		Total (%)
	Male	Female	
*P. perniciosus*	221	120	341 (41.94)
*P. perfiliewi*	135	120	255 (31.36)
*S. minuta parotti*	164	49	213 (26.19)
*P. papatasi*	3	6	3 (0.36)
*P. longicuspis*	1	0	1 (0.12)
Total	524	289	813 (100)

**Table 2:** Detection of TOSV and *L. infantum* infections in field-collected sandflies from the exposure site, 2020.

Date of Collection	Positive Pools	Nbr of Sandflies per Pool (Sex)	PCR *Leishmania* ITS-rDNA	RT-PCR, Nphlebo/A TOSV 2-
7 September	T96	30 (F)	+ (*L. infantum*)	-
	T99	30 (F)	-	+ (TOSV)
	T114	30 (M)	-	+ (TOSV)
	T117	24 (F)	-	+ (TOSV)
21 September	T139	30 (F)	-	+ (TOSV)
	T145	30 (M)	-	+ (TOSV)
31 September	T153	30 (F)	+ (*L. infantum*)	-
8 October	T173	12 (F)	-	+ (TOSV)

M: Males, F: Females, T: sandflies from the field.

**Table 3:** Detection of *L. infantum* and TOSV in *P. perniciosus* fed on dogs.

	Date	Dog A		Dog B		Dog C		Healthy Dog	
Start of dogs exposition	22 July 2020	L. i	TOSV	L. i	TOSV	L. i	TOSV	L. i	TOSV
End of dogs exposition	19 November 2020								
Day 0 post-blood feeding	20 November 2020	-	-	+	-	-	+	-	-
Day 7 post-blood feeding	26 November 2020	-	-	+	-	-	+	-	-

L. i: *L. infantum*. TOSV: Toscana virus.

**Table 4:** Homology and genetic distance between L RNA sequence (201 nt) from TOSV detected in females *P. perniciosus* fed on dog C_7_ and the six TOSV sequences detected in field-collected sandflies.

		1 Dog C_7_	2 T99	3 T114	4 T117	5 T139	6 T145	7 T173
1	Dog C_7_	100/0	0.005	0.000	0.009	0.000	0.000	0.000
2	T99	99.5	100/0	0.005	0.014	0.005	0.005	0.005
3	T114	100	99.5	100/0	0.009	0.000	0.000	0.000
4	T117	99.1	98.6	99.1	100/0	0.009	0.009	0.009
5	T139	100	99.5	100	99.1	100/0	0.000	0.000
6	T145	100	99.5	100	99.1	100	100/0	0.000
7	T173	100	99.5	100	99.1	100	100	100/0

## Data Availability

The data presented in this study are available on request from the corresponding author.

## Supplementary Materials

The following supporting information can be downloaded at: https://www.mdpi.com/article/10.3390/v15041012/s1, Table S1: Detection of TOSV and *L. infantum* infections in all field-collected sandflies from the exposure site, 2020.
