# Supplementary material for: The Role of the Leishmania infantum Infected Dogs as a Potential Reservoir Host for Toscana Virus in a Zoonotic Visceral Leishmaniasis Focus of Northern Tunisia
Source: Viruses. 2023 Apr 20;15(4):1012. doi: 10.3390/v15041012 (PMC10143639; doi:10.3390/v15041012)
Supplement: Supplementary file 1 [file viruses-15-01012-s001.zip › viruses-2293861-supplementary.pdf]

Date collection	Pool number	Number of sandflies	Sex
11.06.2020	T1	1	M
	T2	1	F
	T3	2	M
29.06.2020	T4	27	F
	T5	15	M
	T6	13	M
	T7	10	F
16.07.2020	T8	25	F
	T9	16	M
	T10	9	F
	T11	6	M
	T12	15	F
	T13	11	M
13.08.2020	T14	30	F
	T15	30	M
	T16	30	F
	T17	28	M
	T18	30	F
	T19	30	F
	T20	30	M
	T21	28	F
	T22	30	F
	T23	24	M
	T24	30	F
	T25	30	F
	T26	30	F
	T27	30	F
	T28	30	M
	T29	30	F
	T30	30	M
	T31	30	M
	T32	20	M
	T33	30	F
	T34	30	F
	T35	30	F
	T36	30	M
	T37	30	M
	T38	24	M
	T39	30	F
17.08.2020	T40	30	F
	T41	30	M
	T42	30	F
	T43	30	F
	T44	26	M
	T45	30	F
	T46	23	M
	T47	30	F
	T48	30	F

	T49	30	M
	T50	30	F
	T51	30	M
	T52	30	F
	T53	12	M
	T54	30	F
	T55	30	M
	T56	30	F
	T57	20	M
20.08.2020	T58	30	F
	T59	30	M
	T60	30	F
	T61	30	F
	T62	30	M
	T63	30	F
	T64	30	F
	T65	30	M
	T66	30	M
	T67	30	F
	T68	30	M
24.08.2020	T69	30	F
	T70	30	F
	T71	30	M
	T72	30	F
	T73	30	M
	T74	30	M
	T75	30	F
	T76	21	M
	T77	30	F
	T78	16	M
	T79	30	F
31.08.2020	T80	30	F
	T81	30	M
	T82	30	F
	T83	30	F
	T84	30	F
	T85	30	M
	T86	30	M
	T87	30	M
	T88	30	F
	T89	10	M
7.09.2020	T90	30	F
	T91	30	F
	T92	30	F
	T93	30	M
	T94	30	F
	T95	30	M
	T96	30	F
	T97	30	F
	T98	30	M

	T99	30	F
	T100	30	F
	T101	30	M
	T102	30	F
	T103	30	M
	T104	30	M
	T105	30	F
	T106	30	M
	T107	30	M
	T108	30	M
	T109	30	F
	T110	30	M
	T111	30	M
	T112	30	F
	T113	30	F
	T114	30	M
	T115	30	F
	T116	29	M
	T117	24	F
	T118	30	M
15.09.2020	T119	30	F
	T120	30	M
	T121	30	F
	T122	30	M
	T123	30	F
	T124	30	M
	T125	30	M
	T126	30	F
	T127	30	F
	T128	30	F
	T129	30	M
	T130	30	F
	T131	24	M
	T132	30	F
	T133	30	F
	T134	30	F
	T135	30	M
21.09.2020	T136	30	F
	T137	30	M
	T138	19	M
	T139	30	F
	T140	30	M
	T141	30	F
	T142	30	M
	T143	30	F
	T144	30	M
	T145	30	M
	T146	10	M
31.09.2020	T147	30	F
	T148	30	M

	T149	30	F
	T150	24	M
	T151	30	F
	T152	25	M
	T153	30	F
	T154	30	M
	T155	16	M
	T156	30	F
	T157	30	F
	T158	30	M
	T159	30	F
	T160	30	M
06.10.2020	T161	30	F
	T162	30	F
	T163	30	M
	T164	30	M
	T165	30	F
	T166	30	M
09.10.2020	T167	30	F
	T168	30	F
	T169	30	F
	T170	30	M
	T171	30	F
	T172	30	F
	T173	12	F
	T174	30	M
	T175	30	F
	T176	30	F
	T177	30	M
	T178	30	F
	T179	30	F
	T180	30	F
	T181	30	F
	T182	30	F
	T183	30	F
	T184	30	F
16.10.2020	T185	30	F
	T186	30	F
	T187	30	F
	T188	30	F
	T189	26	M
	T190	30	F
19.10.2020	T191	30	F
	T192	30	F
	T193	30	M
	T194	30	F
	T195	30	M
	T196	30	M
	T197	30	F
22.10.2020	T198	30	F

	T199	30	M
	T200	30	F
	T201	30	F
	T202	30	F
	T203	30	F
	T204	30	F
	T205	30	M
	T206	30	F
	T207	22	M
25.10.2020	T208	29	F
	T209	26	F
31.10.2020	T210	30	F
	T211	30	F
	T212	30	F
	T213	30	F
01.11.2020	T214	30	F
	T215	25	M
	T216	30	F
	T217	30	F
	T218	28	M
	T219	30	F
02.11.2020	T220	6	F
	T221	19	M
03.11.2020	T222	18	M
	T223	15	M
	T224	12	M
	T225	9	M
	T226	3	F
	T227	10	M
	T228	5	F
	T229	2	M
